# How should tracers be injected to detect for sentinel nodes in gastric cancer – submucosally from inside or subserosally from outside of the stomach?

**DOI:** 10.1186/1756-9966-27-79

**Published:** 2008-12-03

**Authors:** Yoshihisa Yaguchi, Takashi Ichikura, Satoshi Ono, Hironori Tsujimoto, Hidekazu Sugasawa, Naoko Sakamoto, Yusuke Matsumoto, Kazumichi Yoshida, Shigeru Kosuda, Kazuo Hase

**Affiliations:** 1 Department of Surgery, National Defense Medical College, 3-2 Namiki, Tokorozawa, Saitama 359-8513, Japan; 2 Department of Radiology, National Defense Medical College, 3-2 Namiki, Tokorozawa, Saitama 359-8513, Japan,3-2 Namiki, Tokorozawa, Saitama 359-8513, Japan

## Abstract

**Background:**

In sentinel node (SN) detection for cases of early gastric cancer, the submucosal dye injection method appears to be more reasonable than the subserosal injection. To compare the two injection methods, we have focused on the rate of concordance between hot nodes (HNs) obtained from the radioisotope (RI) method and green nodes (GNs) obtained from the dye-guided method in addition to the number and distribution of GNs detected, and the sensitivity of metastatic detection.

**Methods:**

The subjects of this study were 63 consecutive patients with gastric cancer (sT1–T2, sN0, tumor diameter ≦ 4 cm) in whom we attempted SN detection using a combination of RI and dye methods. ^99m^Tc-tin colloid was injected a day before the surgery, and indocyanine green was injected either submucosally (n = 43) with endoscopes or subserosally (n = 20) by direct vision.

**Results:**

An average of hot and green nodes (H&G: 4 ± 3 vs. 4 ± 3), hot and non-green nodes (H&NG: 2 ± 3 vs. 1 ± 2), cold and green nodes (C&G: 2 ± 2 vs. 3 ± 4), and the rate of concordance (H&G/H&G + H&NG + C&G: 45 + 27% vs. 48 ± 30%) were not significantly different between the submucosal and subserosal injection methods. The spread of GNs to tier 2 stations (24% vs. 30%) and metastatic detection sensitivity (86% vs. 100%) were also not different between the submucosal and subserosal injection methods.

**Conclusion:**

The tracer injection sites do not have to be limited to the submucosa.

## Background

In recent years, a number of feasibility studies for the sentinel node (SN) concept in gastric cancer have been conducted, and variable results have been reported [1-12]. We have reported that SN biopsy is a useful tool for individualizing surgery for early gastric cancer [12]. Most researchers have used colloid particles labeled with radioisotope and/or dyes as tracers for SN detection. Radioactive tracers must be injected endoscopically into the submucosa preoperatively, since radioisotopes cannot be handled outside of the radiation controlled area under the Japanese law. In the dye procedure, however, there are variations in the method in the form of submucosal injection with endoscopes and subserosal injection of the dye agent from the outside of the gastric wall by direct vision. When a detection procedure is administered for cases of early gastric cancer, submucosal injection appears to be more reasonable, but there are no reliable comparative studies of submucosal injection and subserosal injection for this purpose. Injecting tracers precisely around the tumor, which may be most important to identify true SNs, totally depends on the skill of endoscopists in cases of submucosal injection. On the other hand, subserosal injection from the outside of the gastric wall is easy and accurate as long as the tumor location is identified during surgery. We have used a combination of a radio-guided method using ^99m^Tc-labeled tin colloid and a dye-guided method using indocyanine green (ICG) solution. ^99m^Tc-tin colloid was injected submucosally, and ICG solution was injected either submucosally or subserosally. In this study, we compared the two methods of injection, submucosal or subserosal, focusing on the rate of concordance between hot nodes (HNs) obtained from the RI method and green nodes (GNs) obtained from the dye-guided method. We have also focused on the number and distribution of GNs detected and the metastatic detection sensitivity. Finally, we have weighed the merits of each injection method.

## Subjects and methods

The subjects of the study were 63 patients with T1-2 gastric cancer with tumor dimension of 4 cm or less and with no apparent lymph node metastasis in whom we attempted the SN detection procedure through the concurrent use of the RI and dye-guided methods during the period from January 2003 to March 2008. Of these, ICG solution was injected submucosally in 43 patients and subserosally in the remaining 20 patients.

We injected 0.5 ml of ^99m^Tc-tin colloid at each of four sites surrounding the tumor with endoscopes a day before the surgery. Immediately following laparotomy, we injected 4 ml of 1.25% indocyanine green solution either into the submucosa surrounding the tumor with endoscopes or into the subserous and muscular layers surrounding the tumor from the outside of the stomach by direct observation. When we decided which of the two injection methods was used, we chose the one with which the tracer would be injected more accurately around the tumor. For example, subserosal injection from the outside of the stomach was chosen with a tumor located on the anterior wall of the stomach, and endoscopic submucosal injection was chosen with a tumor of the upper part of the stomach. We employed submucosal injection with a tumor on the lesser curvature since tracers could be injected directly into lymphatic vessels by subserosal injection. We avoided endoscopic submucosal injection when it was needed to inject the tracer tangentially to the gastric mucosa. In cases of subserosal injection with a tumor that was not palpable, the location of the tumor was identified by intraoperative endoscopy. Beginning 5 minutes after the injection of the dye, we dissected lymph node stations where HNs and/or GNs were distributed as quickly as possible. Then, we added dissection of the remaining lymph node stations, which was required for preoperatively planned dissection. The HNs and GNs were detected on a back table.

The rate of concordance of HNs and GNs was calculated as follows:

Concordance rate = H&G/(H&G + H&NG + C&G),

where H&G = the number of hot and green nodes, H&NG = the number of hot and non-green nodes, and C&G = the number of cold and green nodes.

All data were analyzed using the chi-square test or Mann-Whitney U test. A p value of less than 0.05 was considered significant. SN identification was done under the approval of the Institutional Review Board of the National Defense Medical College, and written informed consent was obtained from every patient.

## Results

There were no significant differences in the clinicopathological characteristics between the 43 patients who received submucosal injection of ICG solution and the 20 patients who received subserosal injection (Table 1). No differences were observed in the numbers of hot and green nodes (H&G), hot and non-green nodes (H&NG), cold and green nodes (C&G), and GNs between the two groups of patients. The percentage of patients in whom GNs were distributed to tier 2 stations did not differ between the two groups either. The concordance rate of HNs and GNs was 45 ± 27% for the cases with submucosal injection and 48 ± 30% for the cases with subserosal injection. There was no significant difference between the two injection methods. When the 52 patients with pathologically T1 tumors were analyzed, no differences were observed in clinicopathological background factors, the numbers of H&G, H&NG, and C&G, or the number and distribution of GNs. The concordance rate was 45 ± 27% for the 38 cases with submucosal injection and 48 ± 30% for the 14 cases receiving serosal injection. There was also no significant difference between the two injection methods (Tables 1, 2).

**Table 1:** Clinicopathological characteristics

	Submucosal injection	Subserosal injection	p value
Number of patients	43 (38)	20 (14)	
Age (mean ± SD)	63 ± 8 (63 ± 8)	61 ± 15 (61 ± 16)	p = 0.61 (0.64)
Sex			p = 0.62 (0.75)
Male	28 (27)	15 (10)	
Female	15 (11)	5 (4)	
Tumor size (mean ± SD)	2.8 ± 1.8 (2.7 ± 1.3)	2.8 ± 1.3 (2.6 ± 1.5)	p = 0.71(0.44)
Histology			p = 0.89 (0.92)
Differentiated	25 (23)	12 (8)	
Undifferentiated	18 (15)	8 (6)	
Depth			p = 0.62 (0.98)
M	18	6	
SM	20	8	
MP	4	4	
SS	1	2	
Lymph node metastasis			
Japanese classification			p = 0.46 (0.84)
N0	36 (32)	13 (11)	
N1	6 (6)	6 (2)	
N2	1 (0)	1 (1)	
UICC classification			p = 0.18 (0.69)
N0	36 (32)	13 (11)	
N1	7 (6)	7 (3)	

Lymphatic invasion			p = 0.68 (0.91)
ly0	27 (27)	8 (8)	
ly1	12 (9)	8 (4)	
ly2	3 (2)	3 (1)	
ly3	1 (0)	1 (1)	

Surgical procedure			p = 0.68 (0.72)
Partial gastrectomy	2 (1)	3 (3)	
Sleeve gastrectomy	13 (12)	8 (8)	
Pylorus preserving gastrectomy	6 (6)	3 (0)	
Distal gastrectomy	13 (12)	6 (3)	
Proximal gastrectomy	6 (5)	0 (0)	
Total gastrectomy	3 (2)	0 (0)	

Mean ± SD

Data for the patients with T1 tumors are shown in parentheses

**Table 2:** Numbers and concordance of hot and green nodes

	Submucosal injection	Subserosal injection	p value
H & G*	4 ± 3 (4 ± 3)	4 ± 3 (4 ± 2)	p = 0.78 (0.91)
H & NG*	2 ± 3 (2 ± 3)	1 ± 2 (1 ± 2)	p = 0.45 (0.57)
C & G*	2 ± 2 (3 ± 3)	3 ± 4 (2 ± 4)	p = 0.95 (0.37)
Concordance of HN and GN	45 ± 27% (45 ± 27%)	48 ± 30% (48 ± 30%)	p = 0.42 (0.33)

GNs median, range*	5, 0–16 (5, 0–16)	6, 1–17 (6, 1–17)	p = 0.55 (0.81)

N2 distribution rate≦	24% (21%)	30% (25%)	p = 0.62 (1)

Mean ± SD

H & G: hot and green nodes. H & NG: hot and non-green nodes. C & G: cold and green nodes. GNs: green nodes.

*Figures are numbers of hot and/or green nodes detected.

Data for the patients with T1 tumors are shown in parentheses

Among all subjects, 14 had lymph node metastasis, 7 of the 43 cases (16%) with submucosal injection and 7 of the 20 cases (35%) with subserosal injection. There was only one patient who showed metastasis not in hot or green nodes but in one of the cold and non-green nodes. ICG solution was injected submucosally, but no GNs were detected in that case (case #5 in Table 3). The remaining 13 patients with positive lymph node metastasis showed metastasis in hot or green nodes. Thus, the sensitivity of metastatic detection was 93% for the entire group of subjects, 86% for patients with submucosal injection, and 100% for patients with serosal injection. There was no significant difference in detection between the submucosal and subserosal injection methods (Table 3).

**Table 3:** Lymph node metastasis according to the distribution of the tracers

	H & G*	H&NG*	C&G*	C&NG*	Concordance of HN and GN	Tumor depth	Tumor size (mm)	Histology
Submucosal injection								
#1	2 (1)	ND	1(0)	22 (0)	67%	SM	44	Differentiated
#2	1 (0)	2 (1)	ND	35 (0)	33%	M	23	Differentiated
#3	3 (2)	1 (0)	2 (0)	39 (2)	60%	SS	50	Differentiated
#4	11 (3)	1 (0)	1 (0)	26 (0)	85%	SM	18	Undifferentiated
#5	ND	2 (0)	ND	16 (1)	0%	SM	25	Undifferentiated
#6	1 (1)	6 (0)	2 (0)	8 (0)	11%	SM	40	Undifferentiated
#7	7 (1)	4 (0)	5 (0)	18 (0)	44%	SM	22	Differentiated
Subserosal injection								
#8	1 (1)	6 (0)	ND	21 (0)	14%	SM	18	Differentiated
#9	6 (3)	2 (0)	ND	48 (2)	75%	SM	11	Differentiated
#10	12 (1)	4 (0)	5 (0)	18 (0)	57%	SS	22	Differentiated
#11	6 (1)	3 (0)	3 (0)	25 (1)	50%	MP	45	Undifferentiated
#12	3 (1)	ND	8 (1)	21 (1)	27%	SS	32	Differentiated
#13	7 (4)	1 (0)	1 (0)	47 (0)	77%	SM	37	Undifferentiated
#14	3 (3)	ND	ND	11 (0)	100%	MP	45	Undifferentiated

*Figures are numbers of isolated lymph nodes with numbers of metastatic nodes in parentheses

ND: not detected

## Discussion

The gastric lymphatic stream is very complicated and has been researched for a long time. It has become clear that there are three lymphatic plexuses in the gastric wall, the submucosal, muscular, and subserosal lymphatic plexuses [13]. A submucosal dye injection appears to be more reasonable in cases of gastric cancer with tumor invasion limited to the mucosa or submucosa. However, lymphatic vessels are connected to each other by a communicating branch in the gastric wall which expands vertically [13]. Thus, tracers injected subserosally may spread in the same way as tracers injected submucosally. Subserosal injection may enable us to inject a tracer precisely around the tumor on the grounds that it is possible to insert a needle straight [14]. In fact, an excellent result has been reported in the feasibility study of the SN concept using serosal injection of ICG solution in patients with early gastric cancer [4].

To determine which method is more efficient for SN identification using ICG solution, submucosal injection or subserosal injection, we focused on the rate of concordance between HNs and GNs, the number and distribution of GNs detected, and the metastatic detection sensitivity. We found no differences in these parameters between submucosal injection and subserosal injection, although it is difficult to draw a conclusion concerning the metastatic detection sensitivity due to small number of patients with metastatic lymph nodes. If the more patients with positive node are enrolled, it might be statistically significant. Some authors were concerned that the injection site of tracers could be inaccurate by the subserosal approach because the primary lesion was not always palpable from the serosal side [2]. In such nonpalpable cases, we used endoscopy to identify the accurate location of the primary tumor, even for subserosal dye injection. We consider such situation is adequate due to inject tracer precisely around the tumor.

Lee et al., comparing the subserosal with the submucosal dye (isosulfan blue) injection method in patients with gastric cancer, found no significant differences between them in detection rates, the mean number of SNs, or the sensitivity of the SN biopsies. They concluded that both injection methods were equally efficient for SN biopsy in patients with gastric cancer, but that the serosal injection method was preferable due to its easy technique and short operation time [15]. Although their results are in accordance with ours, the sensitivity of detecting node metastasis was 45% for submucosal injection method and 61% for subserosal injection in their study, which were considerably low sensitivity rates as compared with our study and other studies reported previously [1,2,4-8,10,12]. We could not find any other studies that compared the two methods, submucosal and subserosal injection of tracers, for detecting SNs in gastric cancer. Choosing appropriate tracers and injecting them accurately around the tumor are essential for identifying SNs in early gastric cancer. We conclude that tracers can be injected either submucosally from inside or subserosally from outside of the stomach, as long as they are injected precisely in the area surrounding the tumor.

## Competing interests

The authors declare that they have no competing interests.

## Authors' contributions

All the authors contributed as mentioned. YY, HS, NS, YM and KY participated in the design and acquisition of data. TI, SO and HT conceived of this study, and participated in the design and coordination. SK gave radiological suggestion to this work and KH acted as moderator of this work.
